# Supplementary material for: Modulating Microglia/Macrophage Activation by CDNF Promotes Transplantation of Fetal Ventral Mesencephalic Graft Survival and Function in a Hemiparkinsonian Rat Model
Source: Biomedicines. 2022 Jun 19;10(6):1446. doi: 10.3390/biomedicines10061446 (PMC9221078; doi:10.3390/biomedicines10061446)

## Slide 1
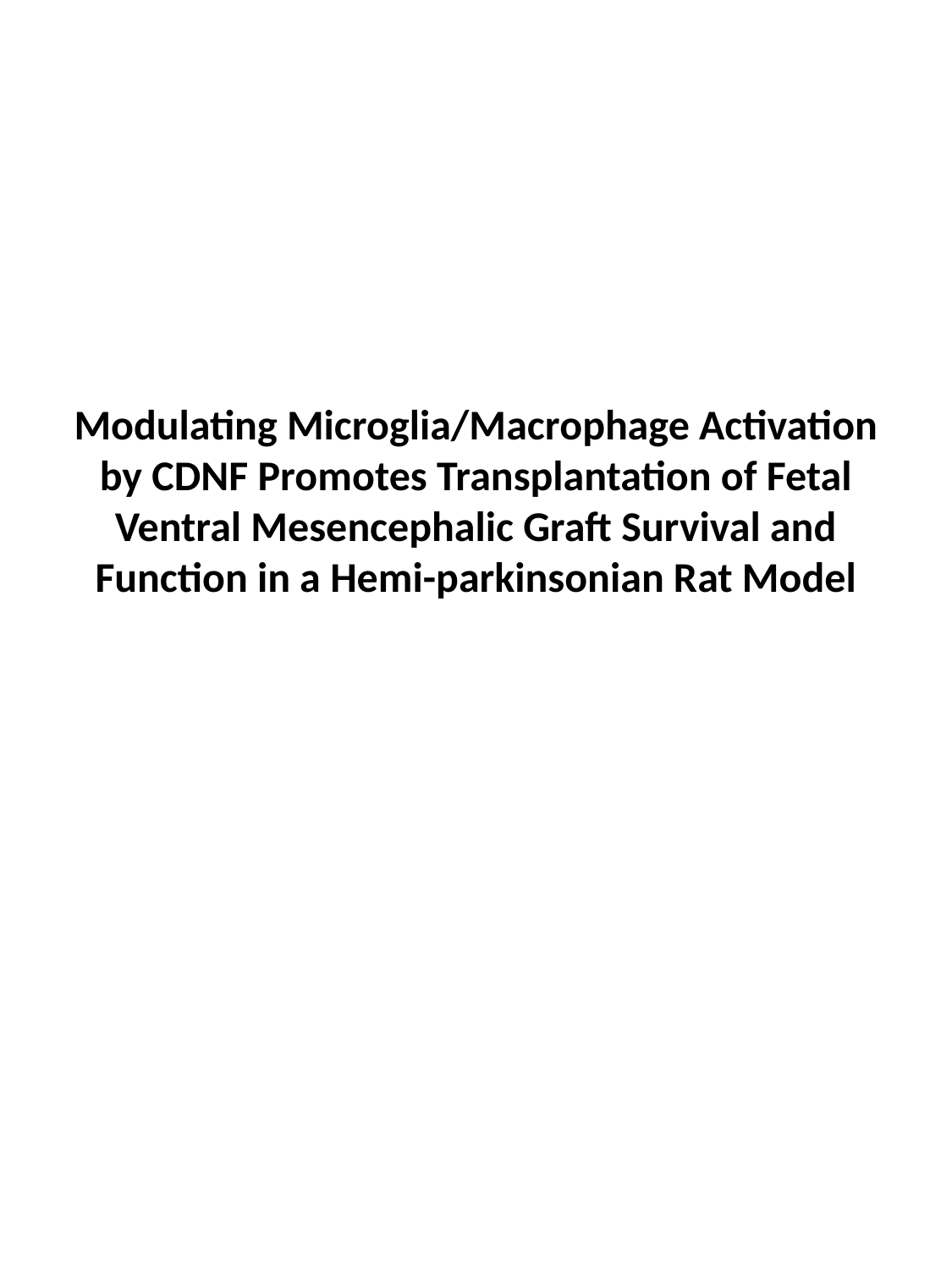

# Modulating Microglia/Macrophage Activation by CDNF Promotes Transplantation of Fetal Ventral Mesencephalic Graft Survival and Function in a Hemi-parkinsonian Rat Model

## Slide 2
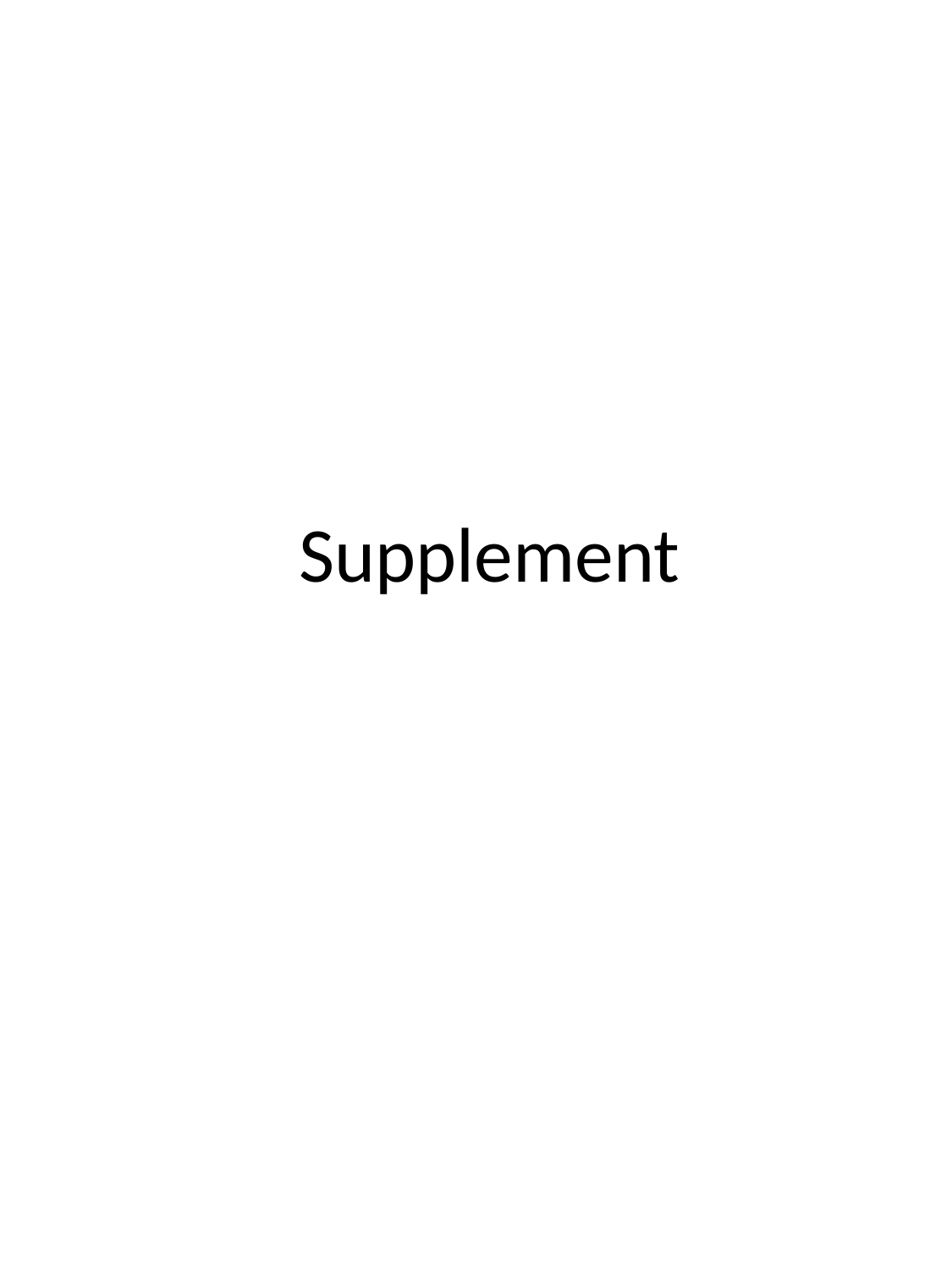

# Supplement

## Slide 3
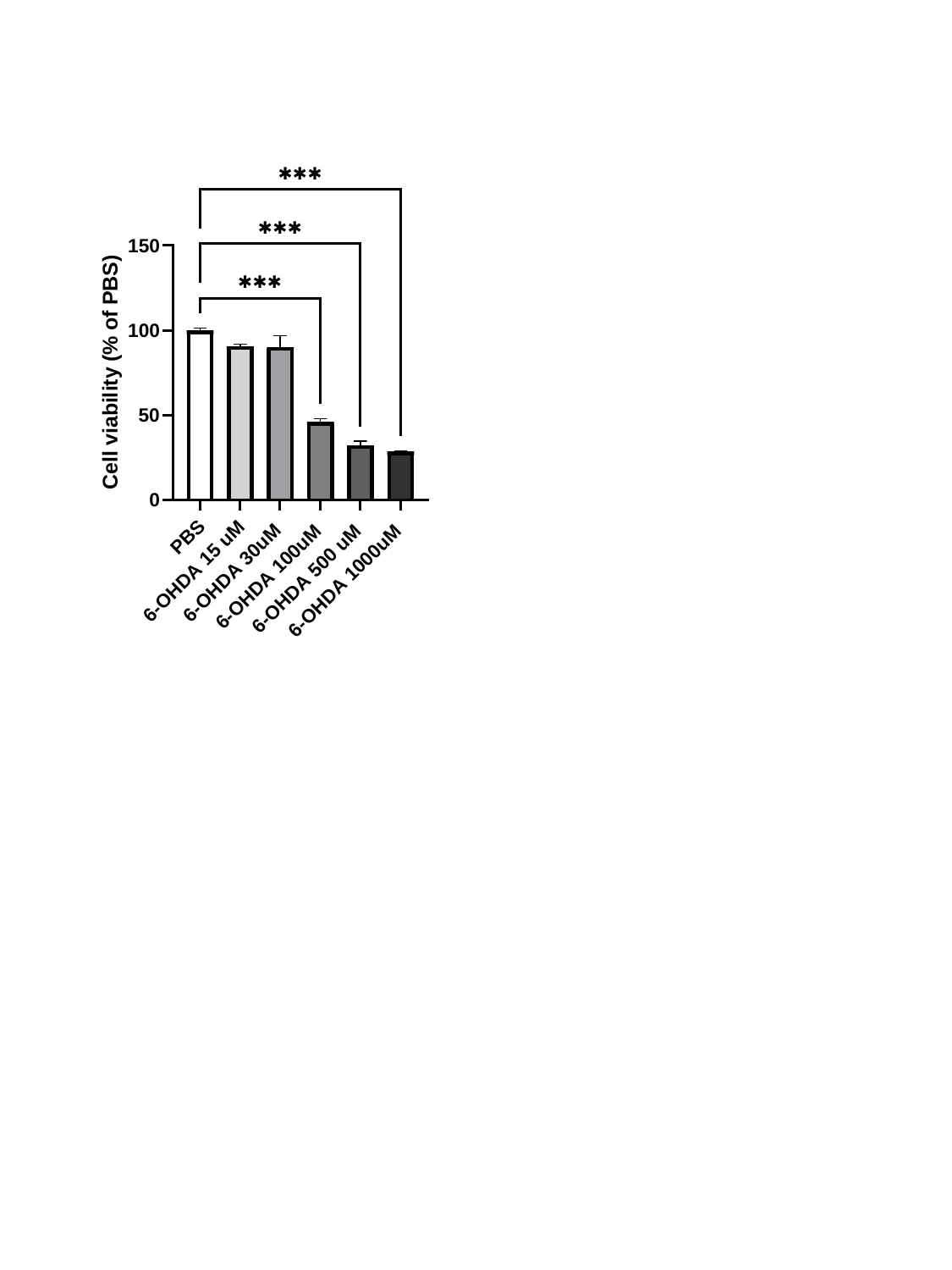

## Slide 4
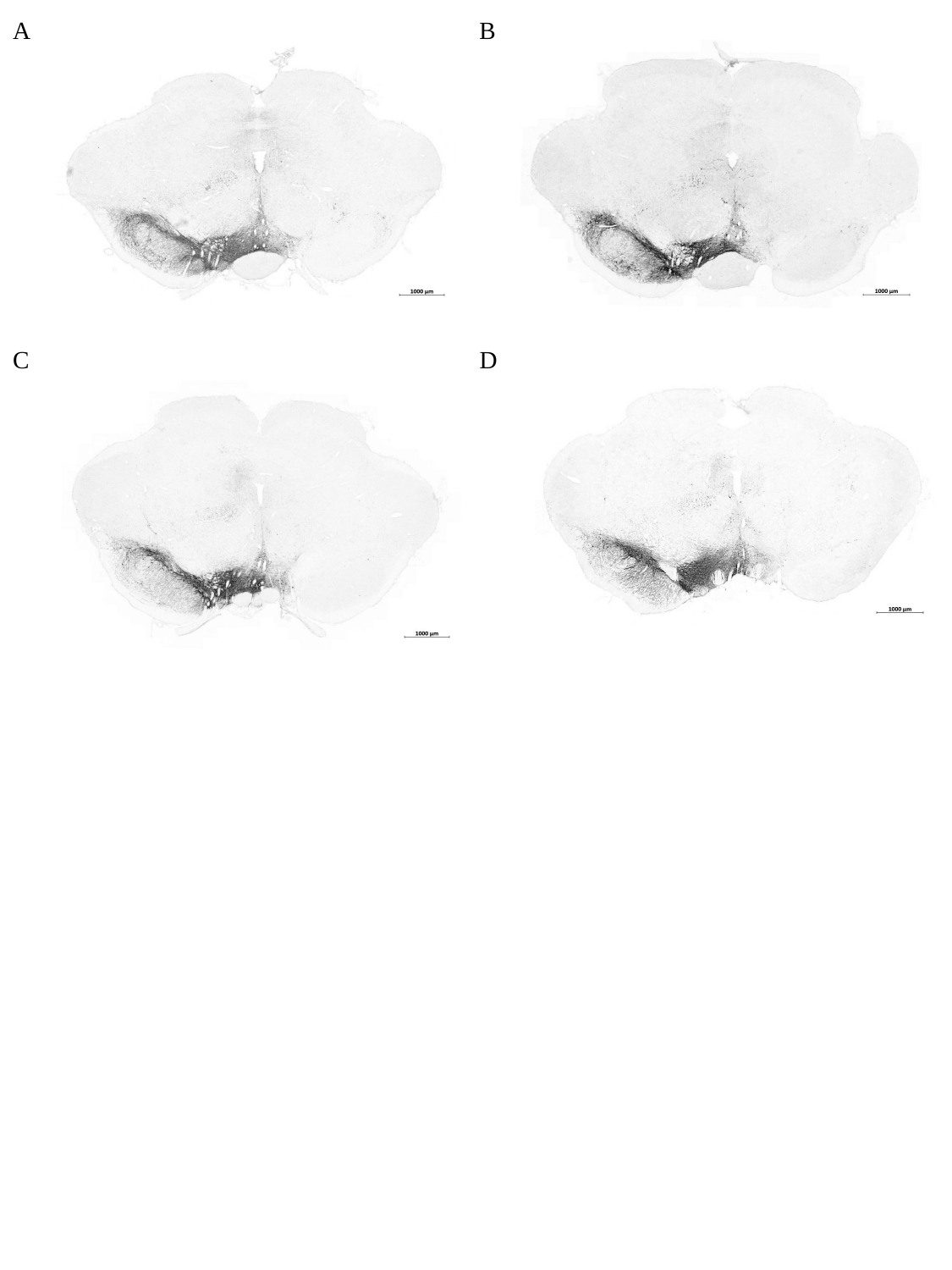

A
B
C
D

## Slide 5
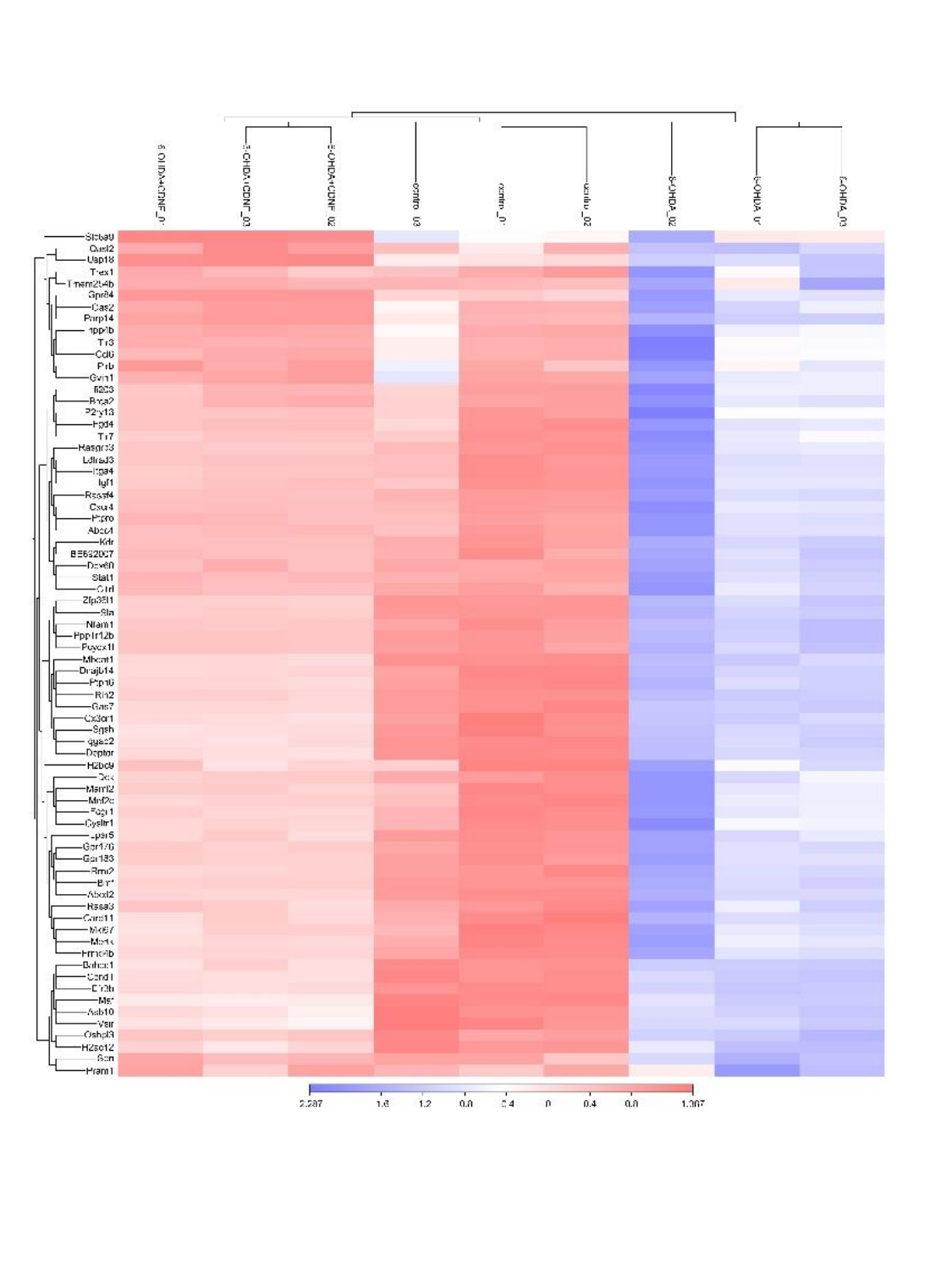

## Slide 6
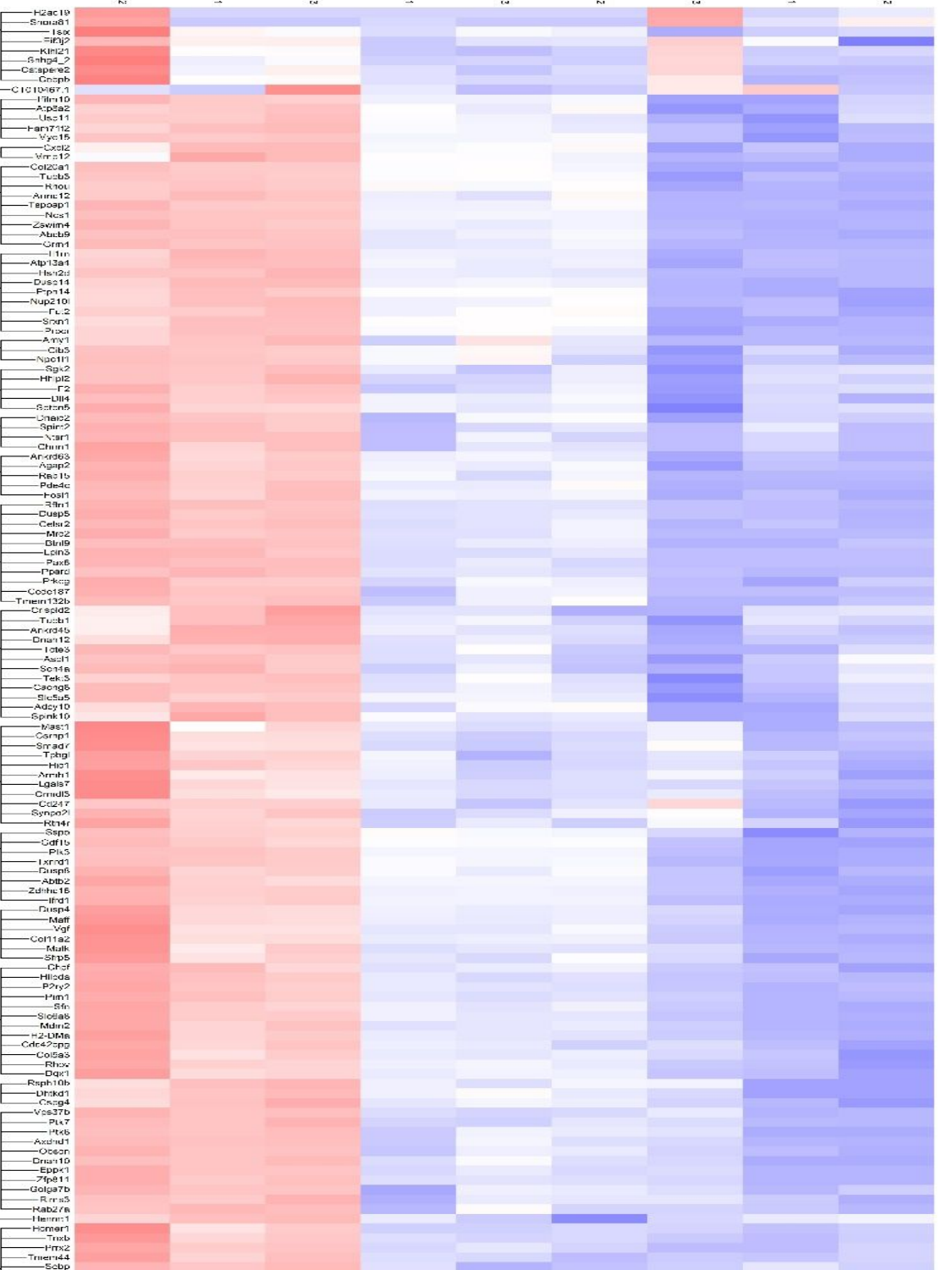

Supplement: Supplementary file 1 [file biomedicines-10-01446-s001.zip › biomedicines-1725888-supplementary.pptx]
